# Notes and News

**Published:** 1895-09-14

**Authors:** 


### NOTES AND NEWS.

(Collected and Communicated.)

Dr. Mitchell has left a legacy of £1,000 to the Walker Hospital, Newcastle, which he built and furnished at his own expense in 1870.

The proposed memorial to Professor Huxley is meeting with much support on all hands. With H.R.H. the Prince of Wales acting as president of the General Committee this could hardly be expected to have any other issue.

Mr. Sinclair, of Glasgow, has made a donation of £500 to the Dunoon Convalescent Homes. Mr. Corbett has presented £1,500 to the Corbett Hospital, Stourbridge, to erect and endow accommodation for children in connection with the institution.

Since the foundation-stone of the Quarrier Consumption Hospitals for Scotland were laid just a year ago, something over £300 only has been subscribed towards the object. One hospital is nearly finished; its cost is calculated at £5,000; of this £3,000 only has been received.

The introductory address of the Medical School of the Westminster Hospital will be delivered by Dr. Monckton Copeman, on Tuesday, October 1st, at four o'clock, and the Right Hon. Viscount Peel (late Speaker of the House of Commons) will preside and distribute the prizes.

The American Medical Press has seized an opportunity of returning the accusations which have often been made against American medical men on the subject of self-advertisement. It is argued that the biographical notices which were published of well-known medical men in the columns of our newspapers at the time of the meeting of the British Medical Association were in reality advertisements, since the material must have emanated from the doctors themselves. We think the impeachment unfair, as such notices, had they been entitled "interviews," would probably have passed unchallenged, even if less accurate and more personal.

We are requested to state that Dr. Ruffer will not be able to deliver the course of lectures, as advertised, at the British Institute of Preventive Medicine, as he has an attack of diphtheritic paralysis, and will not be able to do any work for some time.

During the last week the Dublin hospitals have received the honour of a Viceregal visit. The Earl and Countess of Cadogan, accompanied by a large suite, have inspected a number of the hospitals. Lady Cadogan is a good friend to hospitals, and her interest is of a practical nature; her visit, therefore, is likely to be followed by good results.

The humane will welcome the adoption of the method of slaughtering cattle which was demonstrated at the Guildhall, York, before a meeting of the Church Society for the Promotion of Kindness to Animals. The invention provides that the spinal cord shall be reached promptly and noiselessly by means of a single rifle barrel fitted with a cartridge chamber and simple detonating machine. The company assembled were satisfied that death was produced in a painless manner; and it is to be hoped that so satisfactory a method will be universally adopted in slaughter-houses.

The managers of the Edinburgh Royal Infirmary are extending their appeal for funds to the whole country, the treasurer saying that the institution might be more fitly called the Royal Infirmary of Scotland, considering the number of patients from all parts of the country who are admitted to its wards. He points out that from its very beginning the Royal Infirmary was looked upon more as a national than a local charity, even in the first year of its existence (1729), collections having been received from outlying parts of the country, including one from Evie, in Orkney.

The centenary of the introduction of vaccination will be commemorated in Russia by their National Health Society, who will offer four prizes in connection with the subject, and hold a commemoration meeting on May 14th, 1896. An exhibition of objects relating to vaccination will be arranged also. The possessors of the Jenner relicts, now much scattered, will have an opportunity for bringing them once more before the public. The subjects for the prizes are: "Works on Vaccination," "A History of Vaccination in Russia and Western Europe"; and a Russian translation of Jenner's works, together with his biography, will also be published by the Russian Health Society.

The International Congress of Railway and Steamship Sanitation, to be held at Amsterdam on September 20th and 21st, should prove of much interest. The field is not an unexplored one, but anything approaching a general standard has not hitherto been arrived at. Sanitary rules for navy, merchant, and passenger service vary so largely, and so few statistics are published, that what is essential to health in floating habitations is very little understood, and the subject has not aroused general interest. It is stated that the meeting of railway and ship surgeons is to be especially directed towards the subject of the health of passengers, but a great opportunity will be lost if the occasion is not used to define what is necessary for the well-being of those employed in navigation, who are not in the position to demand what passengers, owing to the competition between steam and railway companies, are mostly able to obtain.

